# The Role of Sonic Hedgehog Signaling in Osteoclastogenesis and Jaw Bone Destruction

**DOI:** 10.1371/journal.pone.0151731

**Published:** 2016-03-23

**Authors:** Tsuyoshi Shimo, Kenichi Matsumoto, Kiyofumi Takabatake, Eriko Aoyama, Yuichiro Takebe, Soichiro Ibaragi, Tatsuo Okui, Naito Kurio, Hiroyuki Takada, Kyoichi Obata, Pai Pang, Masahiro Iwamoto, Hitoshi Nagatsuka, Akira Sasaki

**Affiliations:** 1 Department of Oral and Maxillofacial Surgery, Okayama University Graduate School of Medicine, Dentistry, and Pharmaceutical Sciences, 2-5-1 Shikata-cho, Kita-ku, Okayama, Japan; 2 Department of Oral Pathology and Medicine, Okayama University Graduate School of Medicine, Dentistry, and Pharmaceutical Sciences, 2-5-1 Shikata-cho, Kita-ku, Okayama, Japan; 3 Advanced Research Center for Oral and Craniofacial Sciences, Okayama University Graduate School of Medicine, Dentistry, and Pharmaceutical Sciences, 2-5-1 Shikata-cho, Kita-ku, Okayama, Japan; 4 Department of Orthopaedic Surgery, Thomas Jefferson University, Philadelphia, Pennsylvania, United States of America; University of Oulu, FINLAND

## Abstract

Sonic hedgehog (SHH) and its signaling have been identified in several human cancers, and increased levels of its expression appear to correlate with disease progression and metastasis. However, the role of SHH in bone destruction associated with oral squamous cell carcinomas is still unclear. In this study we analyzed SHH expression and the role played by SHH signaling in gingival carcinoma-induced jawbone destruction. From an analysis of surgically resected lower gingival squamous cell carcinoma mandible samples, we found that SHH was highly expressed in tumor cells that had invaded the bone matrix. On the other hand, the hedgehog receptor Patched and the signaling molecule Gli-2 were highly expressed in the osteoclasts and the progenitor cells. SHH stimulated osteoclast formation and pit formation in the presence of the receptor activator for nuclear factor-κB ligand (RANKL) in CD11b^+^ mouse bone marrow cells. SHH upregulated phosphorylation of ERK1/2 and p38 MAPK, NFATc1, tartrate-resistant acid phosphatase (TRAP), and Cathepsin K expression in RAW264.7 cells. Our results suggest that tumor-derived SHH stimulated the osteoclast formation and bone resorption in the tumor jawbone microenvironment.

## Introduction

Oral squamous cell carcinoma frequently invades into the maxilla and the mandible, and that is associated with a worse prognosis [1]. Medullary invaded oral squamous cell carcinoma has been shown to be an independent predictor of reduced overall and disease-specific survival, and this association appears to result from an increased risk of distant metastatic failure [2]. Bone resection as a treatment leads to the postoperative disruption of speech and swallowing function. Therefore, it is critical that a new approach be generated for the treatment of advanced oral squamous cell carcinoma.

Bone metastatic cancer cells in the bone microenvironment disrupts the bone remodeling cycle and results in bone destruction [3] [4]. A “vicious cycle” between tumor cells and bone microenvironment plays a critical role in tumor-mediated osteolysis [5]. Tumor cells produce osteolytic factors, including parathyroid hormone-related protein (PTHrP) and several interleukins [6]. These factors stimulate the production of the receptor activator of nuclear factor-κB (RANK) ligand (RANKL) in osteoblast lineage cells, and RANKL triggers osteoclast differentiation via binding to RANK on osteoclast precursors [7]. Bone resorption by mature osteoclast releases calcium and growth factors, such as transforming growth factor β (TGF-β) from the bone matrix, and these growth factors further stimulate tumor growth and the secretion of osteolytic factors from tumor cells [3] [6].

Hedgehog signaling contributes to the development [8] [9] and progression of many cancers [10]. During embryonic development, Hedgehog signaling is critical for proper cellular differentiation. The Hedgehog pathway has been associated with organ-specific metastasis to the bone [11]. Paracrine Hedgehog signaling between tumors and stroma has been shown to support malignant growth [12]. Heller et al. [13] and other groups [14] [15] [16] [17] have suggested that Hedgehog-targeted therapeutics may be particularly efficient for tumors that arise within the bone or metastasize to bones due to their effect on host cells within this microenvironment [18]. Recently, we have revealed that SHH stimulates osteoclast differentiation by upregulating RANKL expression in bone stromal cells and osteoblasts in the presence of PTHrP [19] [20]. It is still unclear, however, how Hedgehog signaling participates in osteoclastic bone resorption by oral squamous cell carcinoma cells. In the present study, we analyzed the effect of SHH expression on advanced oral squamous cell carcinomas and how hedgehog signaling was involved in osteoclastic bone resorption associated with tumor invasion.

## Materials and Methods

### Histochemical and immunohistochemical analysis of surgically resected samples

The study was approved by the Ethical Committee of the Okayama University Graduate School of Medicine, Dentistry, and Pharmaceutical Sciences (protocol number: 1949), including use of an advertisement poster in place of patient consent. Written consent was not acquired for this retrospective study. The authors had access to patients’ records prior to data anonymization. All the patients were examined and treated at Okayama University Hospital (Okayama, Japan) between 2000 and 2010, and the diagnosis was clinicopathologically confirmed aggressive invasive phenotype of lower gingival squamous cell carcinoma (n = 10). The surgically resected mandibles were collected as part of routine care by the authors. No patient had received chemotherapy and/or radiation therapy before surgery. All patients’ records/information were anonymised and de-identified prior to analysis. Sections from the deepest part of the invasion and the boundary between the tumor and the bone were evaluated primarily by light microscopic observation. The sections were deparaffinized, and then autoclaved in 0.2% citrate buffer for 15 min for antigen retrieval. Sections were incubated with 3% hydrogen peroxide for 30 min to block endogenous peroxidase activity. A primary anti-SHH (rabbit IgG), anti-Gli-2 (rabbit IgG) (Cell Signaling Technology, Danvers, MA), anti-Patched1 (rabbit IgG, Proteintech, Chicago IL) and anti-CD68 (mouse IgG, Dako, Glostrup, Denmark) was used for the immunohistochemical analysis. The specimens were incubated with a 1:100 dilution of the antibody overnight at 4°C, followed by 3 washes with TBS. The slides were then treated with a streptoavidin-biotin complex (Envision System Labeled Polymer, horse radish peroxidase (HRP); Dako, Carpinteria, CA) for 60 min at a dilution of 1:100. The immunoreaction was visualized using a 3,3'-diaminobenzidine (DAB) substrate-chromogen solution (Dako Cytomation Liquid DAB Substrate Chromogen System, Dako), and counterstaining was performed with hematoxylin. Finally, the sections were immersed in an ethanol and xylene bath and mounted for examination.

### Cell lines and culture conditions

The murine macrophage RAW264.7 cells from the American Type Culture Collection (ATCC) were cultured in Dulbecco's Modified Eagle’s Medium (DMEM) supplemented with 10% heat-inactivated fetal bovine serum (FBS). CD11b^+^ bone marrow cells were cultured in α-Modified Eagle’s Medium (αMEM) with 10% FBS. Both cell types were cultured in an atmosphere of 10% CO_2_ at 37°C.

### Animals and Purification of osteoclast progenitors

Animals were housed in cages under pathogen-free conditions with humane care and anesthesia was performed using pentobarbital 150 mg /kg I.P. to minimize suffering. The experimental protocols for CD11b^+^ osteoclast progenitor cells from C57BL/6 mice (Charles River) was approved by the Ethics Review Committee for Animal Experimentation of the Okayama University Graduate School of Medicine, Dentistry and Pharmaceutical Sciences (protocol number: 2010291), and the animals for floxed Smoothened mice (*Smotm*^*2Amc/J*^, The Jackson Laboratory) was approved for IACUC (Institutional Animal Care and Use Committee) of the Thomas Jefferson University (Authors' Former Institute). CD11b^+^ osteoclast progenitor cells were prepared from 5-week-old male C57BL/6 mice or floxed Smoothened mice. Bone marrow cells were washed twice in 20 ml of ice-cold phosphate-buffered saline (PBS) supplemented with 0.5% bovine serum albumin (Sigma, St. Louis, MO) and 2 mM EDTA (Sigma). The cell pellet was resuspended in 80 μl of buffer A per 10^7^ cells, and was incubated with 20 μl of anti-CD11b magnetic microbeads (Miltenyi Biotec Inc., Auburn, CA). The labeled cells were washed, resuspended in 500 μl of buffer A per 10^8^ cells, and applied onto MD depletion column (Miltenyi Biotec Inc., Bergisch Gladbach, Germany) placed in the magnetic field of a MidiMACS separation unit (Miltenyi Biotec Inc.). After washing, we removed the column from the separation unit and eluted CD11b^+^ cells. To study the direct role of hedgehog signaling in osteoclastogenesis, we removed smoothened gene in CD11b^+^ positive osteoclast precursor cells from floxed Smoothened mice by infecting Cre-recombinase encoding adenovirus (Ad-CMV-iCre, Vector Biolab, Malvem, PA). Briefly, freshly isolated CD11b^+^ positive cells were incubated with 100 pfu of Ad-CMV-Cre or control Ad-CMV-GFP (Vector Biolab) for 1 h in ice. Cells were resuspended every 15 min and then seeded onto the culture plates. Medium was replaced the next day. Adenovirus infection was confirmed at 70–90% 72 h after infection under fluorescence microscopy.

### Immunoblot analysis

RAW264.7 cells were rinsed once with ice-cold phosphate buffered saline (PBS) and lysed in an ice-cold lysis buffer (50 mM Tris-HCl, pH 7.4, containing 150 mM NaCl, 1% Triton X-100, 1% NP-40, 10 mM NaF, 100 mM leupeptin, 2 mg/ml aprotinin, and 1 mM phenylmethyl sulfonyl fluoride). Cell lysates containing 10 μg of total protein in a lysis buffer were electrophoresed in 12% sodium dodecyl sulfate polyacrylamide gel electrophoresis (SDS-PAGE) gels and the proteins were transferred to nylon membranes (Immobilon-P; Millipore Co.). The membranes were incubated with primary and secondary antibodies according to the ECL chemiluminescence protocol (RPN2109; Amersham Biosciences, Buckinghamshire, UK) to detect secondary antibody binding. Anti-ERK1/2 (rabbit IgG), pERK1/2 (Thy202/Tyr204, rabbit IgG), p38 (rabbit IgG), p-p38 (Tyr180/Tyr182, rabbit IgG) were purchased from Cell Signaling Technology Inc. (Danvers, MA). NFATc1 (mouse IgG), Cathepsin K (rabbit IgG), tartrate-resistant acid phosphatase (TRAP) (rabbit IgG) and actin (goat IgG) antibodies were purchased from Santa Cruz Biotechnology (Santa Cruz, CA) primary antibodies were used at a 1:200 dilution, and horseradish peroxidase-conjugated goat anti-rabbit antibodies or goat anti-mouse IgG were used as the secondary antibodies at a 1:1000 dilution. Image-J software (NIH, Bethesda, MD) was used to quantify band intensities on Immunoblots and the statistical analysis was performed by Bonferroni multiple comparisons tests.

### TRAP staining and osteoclast activity assay

RAW264.7 murine macrophage cells were treated with 50 ng/ml of recombinant mouse RANKL (PEPROTECH EC, London, UK) for 5 days CD11b^+^ bone marrow cells and bone marrow macrophages [21] were cultured with 30 ng/ml M-CSF, (R&D System, Minneapolis, MN) and 50 ng/ml RANKL in the presence or absence of SHH (R&D System, Minneapolis, MN) or Cyclopamine (Toronto Chemical Inc., Miltenyi Biotec Inc., Auburn, CA) for 8 days. The complete medium was changed every 2 days. The cells were then fixed and stained for TRAP (Sigma) and the number of TRAP-positive multinucleated cells (nuclear number >3) in each well was counted. For the osteoclast activity assay, CD11b^+^ bone marrow cells were seeded on dentin slices that had been placed in 96-well plates (1 slice/well) and were cultured for 5 days in αMEM containing RANKL (50 ng/ml) and M-CSF (30 ng/ml). Then the medium was changed to αMEM with or without RANKL (50 ng/ml) and SHH (500 ng/ml), and the cells were incubated for 7 days. A scanning electron microscope (SEM, VE9800; Keyence, Osaka, Japan) was used to examine the ultrastructure of the dentin slices. The area of pits on the slices was quantified by imaging analysis (Lumina Vision; Mitani Corporation, Osaka, Japan). The results were expressed as the mean ± SD of three cultures.

### Microarray analysis

RAW264.7 cells were treated with 50 ng/ml RANKL for 3 days, and then cultured with or without 5 μM smoothened agonist (SAG) (Merck Millipore, Parmstadt, Germany) for 24 h. After the treatment with SAG, total RNAs were isolated using an miRNeasy Mini Kit (Qiagen, Valencia, CA). Genome-wide gene expression analysis was performed using SuperPrint G3 Mouse GE 8x60K 2 color (Agilent Technologies, Santa Clara, CA) according to the manufacturer’s protocol. The arrays were scanned and digitized by Agilent Feature Extraction 10.7.3.1 software (Agilent Technologies). The data was analyzed by GeneSpring GX v.13 (Agilent Technologies).

### Quantitative real-time PCR analysis

Total RNA was isolated according to the manufacturer’s protocol (RNeasy Minikit, Qiagen). After RNA reverse transcription, 1 μl of each cDNA preparation was PCR-amplified using the set of primers described below. The tachykinin receptor 3 primmer: 5’-TTGCAGTGGACAGGTATATG-3’ and 5’-GATGTGGTGGAGGCAGATTT-3’, NFATc1 primer: 5’- CAAGTCTCTTTCCCCGACATC-3’ and 5’- CTGCCTTCCGTCTCATAGTG-3’. The condition of quantitative real-time PCR (qPCR) were as follows: 40 cycles of 95°C for 30 seconds 60°C for 30 seconds, and 72°C for 30 seconds for tachykinin receptor 3 or 37 cycles of 95°C for 5 seconds and 62°C for 30 seconds for NFATc1. Melting curves were obtained, RT-qPCR was performed on the chromo4 (MJ Research, Waltham, MA, USA) using a SsoAdvanced Universal SYBR Green Supermix (Bio-Rad Laboratories, Inc. Philadelphia, PA), and the data was analyzed by using ΔΔCT methods.

### Statistical analysis

Data were analyzed by using the unpaired Student’s t-test for analysis of two groups, Fisher’s protected least significant difference (Fisher’s PLSD) and Bonferroni for analysis of multiple groups. Results were expressed as the mean ± S.D. p < 0.05 was considered statistically significant.

## Results

### SHH expression in osteolytic mandibular squamous cell carcinoma

**Fig 1:**
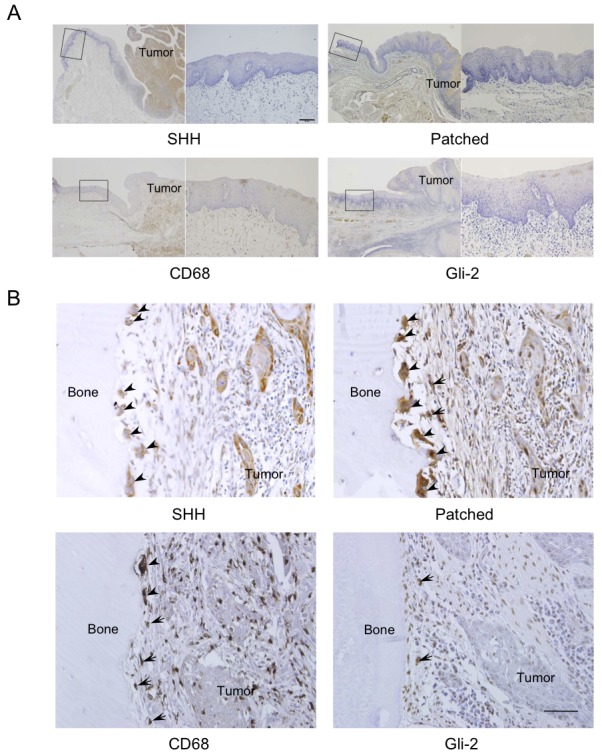
Immunohistochemical staining for Sonic hedgehog (SHH), Patched, CD68 and Gli-2 in normal epithelium and osteolytic mandibular squamous cell carcinoma. (A) SHH, Patched, CD68 and Gli-2 expression in the normal epithelium at the edges of area were the mandible was resected. Each right photo is a magnification of the rectangle delimited area of normal epithelium in the corresponding left photo. Scale bar, 50 μm. (B) SHH, Patched, CD68 and Gli-2 expression in a resected mandible. Scale bar, 50 μm. Arrow: osteoclast. Arrowhead: osteoclasts progenitor cell.

Fig 1 shows representative microscopic images of invasive bone destruction observed in a patient with oral squamous cell carcinoma in the mandibular region. SHH, Patched, CD68 and Gli-2 were not detected in the normal lower gingival epithelium (Fig 1A). SHH was highly expressed in tumor cells that had invaded the bone matrix, and weakly expressed in osteoclasts (Fig 1B). On the other hand, the hedgehog receptor Patched was highly expressed in the osteoclasts and the progenitor cells (Fig 1B). CD68 was detected in both osteoclasts and progenitor cells (Fig 1B). Gli-2 was expressed in the progenitor cells, and weakly expressed in tumor cells (Fig 1B). We analyzed additional 9 cases of mandibular squamous cell carcinoma with invasive bone destruction and observed (i) intense SHH immunoreactivity in tumor cells, (ii) intense Patched and Gli-2 immunoreactivity in osteoclasts and progenitor cells in all cases (not shown).

### Effects of hedgehog proteins on osteoclast formation and resorption

**Fig 2:**
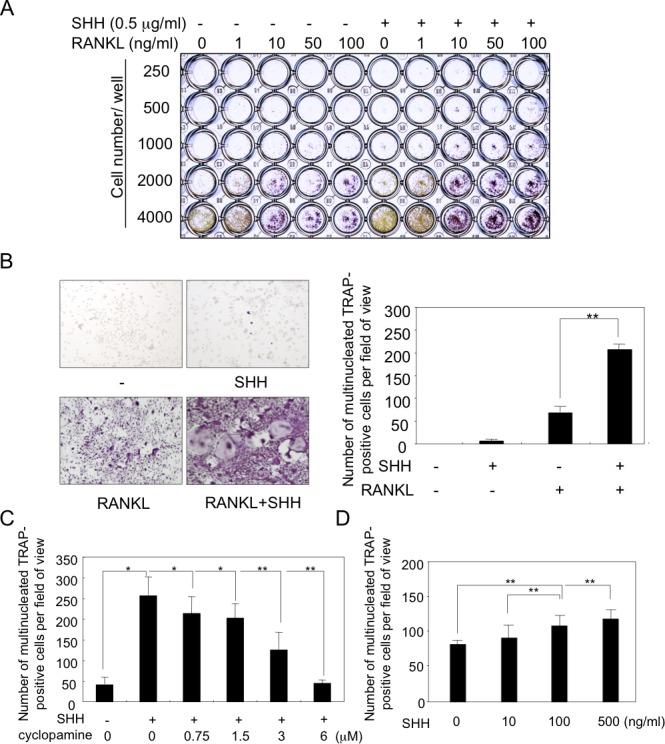
Hedgehog stimulated osteoclast formation of RAW264.7 cells, CD11b^+^ cells in bone marrow, and bone marrow macrophages. (**A**) RAW264.7 cells were inoculated onto a 96-well plate at different cell densities and then treated with increasing concentrations of RANKL +/- SHH for 6 days. Osteoclast formation was evaluated by TRAP staining. (**B and C**) CD11b^+^ cells isolated from the bone marrow of 5-week-old mice were cultured in the presence of 30 ng/ml M-CSF and 50 ng/ml RANKL with or without 500 ng/ml SHH in 96-well plate for 6 days. Cultures were also treated with or without SHH and hedgehog inhibitor cyclopamine for 6 days (C). Bone marrow macrophages were cultured with indicated amount of SHH in the presence of 30 ng/ml M-CSF and 50 ng/ml RANKL in 48-well plate for 7 days. TRAP-positive multinucleated cells (nuclear number > 3) were counted as osteoclasts. The data from a typical experiment are presented. Data are shown as the mean ± SD. Statistically significant differences (*P < 0.05, **P < 0.01) between the indicated groups are marked by asterisks.

**Fig 3:**
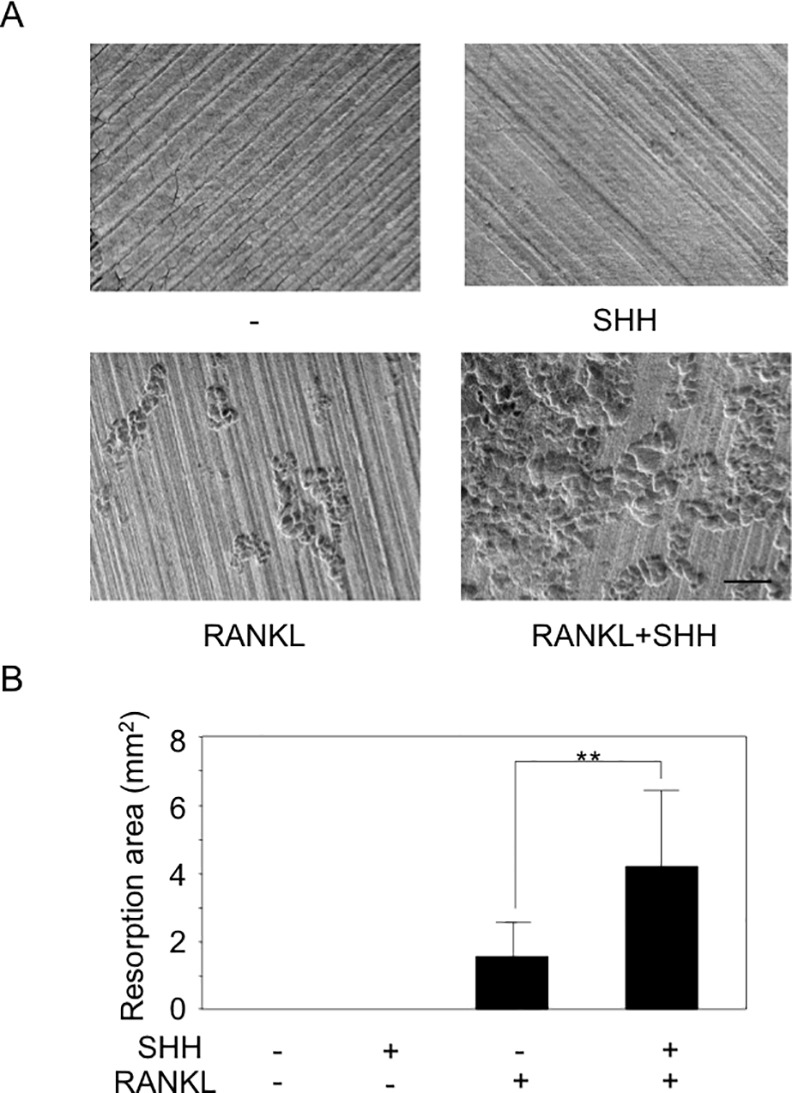
SHH stimulated bone resorptive activity of mature osteoclasts. (**A**) Bone resorptive activity of mature osteoclasts differentiated from CD11b^+^ bone marrow cells were evaluated in a dentin slice. The data from a typical experiment are presented. Similar results were obtained in three separate experiments. (**B**) The resorbed areas on the slides were observed under a microscope and measured. The data from a typical experiment are presented. Data are shown as the mean ± SD. Statistically significant differences (**P < 0.01) between the indicated groups are marked by asterisks. Scale bar, 100 mm.

Next, we studied the effect of hedgehog on osteoclastogenesis. To elucidate the effect of SHH in osteoclast formation, RAW264.7 murine macrophage cells, known as osteoclast precursor cells, were inoculated onto 96-wells plates at different cell densities and then treated with increasing concentrations of RANKL with or without SHH for 6 days. The concentration of SHH for osteoclastogenesis was determined 500 ng/ml by the previous our studies [19], and the osteoclast formation was evaluated by TRAP staining. SHH alone did not affect osteoclastogenesis, but greatly enhanced RANKL-induced osteoclastic cell differentiation (Fig 2A). Next, we evaluated the effect of hedgehog on CD11b^+^ osteoclast precursor cells. CD11b^+^ bone marrow cells were maintained in αMEM containing 10% FBS and 30 ng/ml M-CSF, and treated with or without 50 ng/ml RANKL and 500 ng/ml SHH for 6 days. The cells were then fixed and stained for TRAP activity, and TRAP cells containing 3 nuclei were counted as osteoclasts by microscopy (Fig 2B). As seen in RAW264.7 cells, treatment with SHH alone did not induce osteoclast formation compared to the control values in untreated cultures. As expected, RANKL treatment did induce formation of TRAP-positive and a small number of multinucleated osteoclastic cells. Strikingly, massive osteoclastogenesis was significantly observed following co-treatment with RANKL and SHH (Fig 2B). The data indicate that SHH acts directly on osteoclast progenitor cells and stimulates RANKL-induced osteoclastogenesis. To analyze the effect of SHH on osteoclastogenesis, CD11b^+^ bone marrow cells were treated with or without 500 ng/ml SHH and the hedgehog signaling inhibitor cyclopamine for 6 days. Treatment with cyclopamine significantly blocked the SHH-induced increase in osteoclastogenesis and brought it down to the basal level at 6 μM (Fig 2C). We also confirmed the significant dose dependent effect of SHH on the osteoclastogenesis by using primary isolated bone marrow macrophages (Fig 2D). To further confirm the role of SHH in osteoclast differentiation, we isolated CD11b^+^ bone marrow cells from floxed Smoothened mice, infected cells with either Cre recombinase encoding adenovirus or control adenovirus, and then compared the effect of SHH on RANKL-induced osteoclastogenesis (S3 Fig). As expected, stimulating effects of SHH on RANKL-induced osteoclastogenesis were almost completely abolished by ablation of Smoothened (S3 Fig). Then, we analyzed the effect of SHH on the bone resorption activity of mature osteoclasts. An osteoclast activity assay using CD11b^+^ bone marrow cells showed that SHH alone did not stimulate osteoclast resorption, but significantly enhanced the RANKL-induced bone resorptive activity of osteoclasts (Fig 3A and 3B).

### Hedgehog signaling in osteoclast differentiation

**Fig 4:**
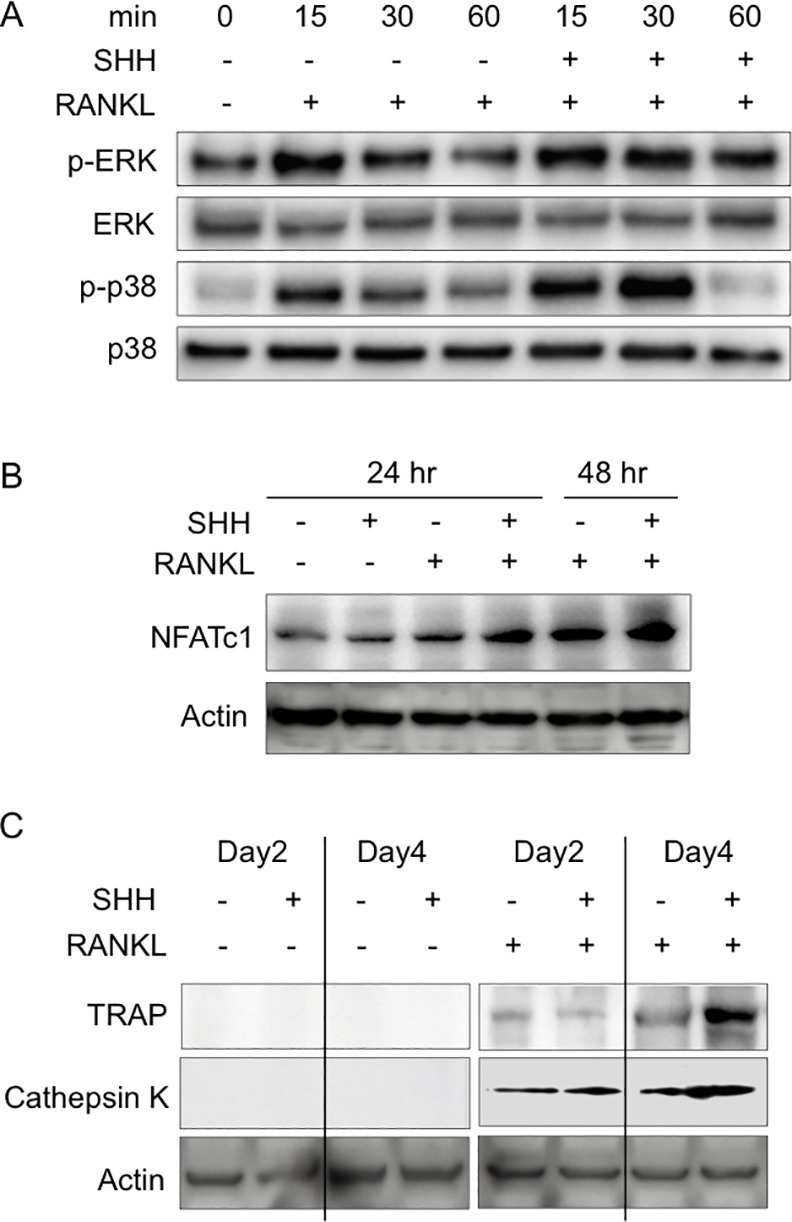
Effects of SHH on MAP kinase activity and osteoclast differentiation. (**A**) Detection of p-ERK, ERK, p-p38, and p38 by immunoblot analysis after RANKL (50 ng/ml) in murine macrophage RAW264.7 cells with or without SHH (500 ng/ml). (**B**) Immunoblot analysis of NFATc1 in RAW264.7 cells 0–48 h after RANKL (30 ng/ml) or SHH (500 ng/ml) treatment. (**C**) The mature osteoclast-differentiated cells were treated with SHH (500 ng/ml) or RANKL (30 ng/ml). Protein expression of TRAP and Cathepsin K was analyzed by immunoblot analysis. Similar results were obtained in three different experiments.

To define the molecular mechanisms of the SHH effects on osteoclast formation, we examined the effects of SHH on the signaling pathways in RAW264.7 cells. RANKL stimulated the transient ERK and p38 phosphorylation at 15 min (Fig 4A). On the other hand, treatment of SHH with RANKL increased the sustained and synergistically promoted ERK phosphorylation for 30 and 60 min (P < 0.05) and transient p38 phosphorylation for 15 and 30 min (P < 0.01) compared with the single treatment of RANKL (Fig 4A). Total ERK and p38 levels remained constant after the treatments (Fig 4A). NFATc1 is a master regulator of RANKL-induced osteoclast formation and herein we investigated the effect of SHH on the NFATc1 expression with or without RANKL. Our results showed that the expression of NFATc1 in the presence of RANKL and SHH was significantly higher than in the presence of RANKL alone for 24 and 48h (Fig 4B). Immunoblot analysis showed that the TRAP and Cathepsin K protein expressions induced by RANKL in RAW264.7 cells were stimulated by SHH treatment for 4 days (Fig 4C). To confirm whether RAW264.7 cells and CD11b^+^ cells share similar mechanisms for SHH’s effect on osteoclast formation, Immunoblot and qPCR analysis were performed. SHH with RANKL synergistically promoted ERK and p38 phosphorylation for 30 and 60 min compared with the single treatment of RANKL in CD11b^+^ cells (S4A Fig). SHH significantly upregulated the relative NFATc1 mRNA levels in the presence of RANKL in CD11b^+^ cells (P < 0.01, S4B Fig).

### Hedgehog signaling is involved in osteoclast differentiation and activation genes

Since SHH appears to play an important role in the differentiation and activation of osteoclasts, we compared the endogenous expression levels of genes between vehicle- or SAG-treated RAW264.7 cells by microarray analysis (S5 Fig). When compared with the vehicle-treated samples, SAG-treated samples showed an elevation of various pathways, most of which were G protein-coupled receptor (GPCR) pathways (S1 Fig). S2 Fig shows the genes that were changed more than three times relevant to the top 3 biological pathways depicted in pathway analysis in S1 Fig. The expression of tachykinin receptor 3 showed the highest upregulation, 9.8-fold over the control level, in the non-odorant GPCR pathways (S2 Fig). The expression of the chemokine receptor 3 (CCR3) gene in the GPCR2 class A rhodopsin-like pathways and olfactory receptor 498 in odorant GPCRs were also upregulated compared to the control levels (S2 Fig).

## Discussion

In previous studies, we reported on the ability of paracrine hedgehog signaling to increase oral squamous cell carcinoma growth in bone through the indirect actions of osteoclastogenesis on bone marrow stromal cells with osteoclast progenitor cells [19]. In the study, we demonstrated that SHH stimulated osteoclast formation by upregulating the production of RANKL in bone marrow stromal cells. In addition to the increased expression of RANKL by SHH recreated by tumor cells, we previously observed a cooperative effect of the PTHrP pathway in osteoblasts on osteoclastogenesis in the context of SHH treatment [20]. Further, our data in this study suggest that paracrine hedgehog signaling can increase oral squamous cell carcinoma growth through direct actions on osteoclast progenitor cells. SHH knockdown in SAS cells tumor engrafts attenuated the recruitment of new vasculature to the tumor, suggesting the involvement of SHH in tumor-associated angiogenesis [19]. Gli-2 expression was recognized in the microvasculature in the tumor bone microenvironments of resected samples (data not shown). Gli-2 expression in tumor cells has been associated with aggressive biological phenotypes in oral squamous cell carcinoma patients and may be used as a biomarker to predict clinical outcome [22]. The tumor growth mechanism by hedgehog signaling has been thought to be primarily mediated through an autocrine loop that might be required for proliferation and survival of oral squamous cell carcinoma cells [23]. In this study, Gli-2 was barely expressed in tumor cells, suggesting that a high level of the ligand SHH can not respond to it in an autocrine fashion. We hypothesized that the production of SHH by tumor cells would promote a paracrine signaling pathway and stimulate hedgehog signaling in bone marrow host cells. Therefore, targeting hedgehog signaling within osteoclasts and the progenitor cells would effectively block the supply of growth factors for tumor progression.

In the present study, the osteoclasts expressed patched1 and hedgehog signaling in oral squamous cell carcinoma cells in the jaw. The function of hedgehog signaling in osteoclasts is uncertain. Heller et al. also reported that disruption of hedgehog signaling inhibited osteoclast size and number by transducing smoothened fl/fl bone marrow macrophages with a lentivirus expressing Cre-recombinase [13]. We also observed a decrease in osteoclasts number after smoothened excision compared to GFP infected control cells. LDE225, smoothened antagonist, reduced the hedgehog target gene Gli1 and key genes involved in osteoclast differentiation (NFATc1 and Cathepsin K) [13]. Moreover, blockade of Shh in SAS cells decreased tumor growth and osteoclast number in a tibial metaphysis mouse model [19]. These results are agreement with our data that SHH increased protein levels of NFATc1, TRAP and Cathepsin K. RANKL signaling triggers osteoclasts differentiation, Binding of RANKL to its receptor, RANK, rapidly activates ERK and p38 MAP kinases. ERK and p38 MAP kinases are essential for the differentiation and activation of osteoclasts [24]. The activated MAP kinases then lead to the stimulation of NFATc1 [24]. On the other hand, SHH signaling pathway is closely associated with ERK1/2 cascade, which is the downstream effector of many important growth factors signaling pathways, and is the core of the signaling networks involved in cell proliferation and differentiation [25] [26]. p38 MAPK activation is proximal to smoothened, and that a subset of receptors is appropriately compartmentalized for signal activation via direct SHH action [27]. Our data suggested that SHH assist the upregulation of the NFATc1 expression via activation of ERK and p38, which contribute of RANKL-induced osteoclastogenesis. To identify the molecular changes that occur when osteoclasts are activated hedgehog signaling, microarray analysis were performed. Pathway analysis revealed that most of the pathways were involved GPCR-related genes. GPCR represent the largest family of membrane receptors. Analysis of the human genome has revealed that more than 800 genes encode these 7 trans-membrane receptors [28]. GPCR stimulation regulates actin remodeling, the activity of ion transport proteins, and cell migration [29] [30] [31]. GPCR can induce signaling events through GRKs by a mechanism involving MAPK [29]. GRK2 and p38 MAPK are regulators of SHH-dependent signaling and gene regulation [27]. These functions are closely related to the multinucleation of osteoclasts, formation of the sealing zone and bone resorption. Tachykinin receptor 3 in non-odorant GPCRs is the most upregulated gene stimulated by hedgehog signaling. The factor is the receptor of Neurokinin B, which activates osteoclast formation and bone resorption [32]. CCR3 is the most upregulated gene in the GPCR2 class A rhodopsin-like group. CCR3 is expressed in osteoclast precursors and promotes the differentiation of osteoclast precursors into mature osteoclasts [33]. We focus on the tachykinin receptor 3 gene which is most upregulated by SAG treatment, it has been confirmed that tachykinin receptor 3 was upregulated by the treatment of 1 μM SAG and 500 ng/ml SHH about 4.90 and 6.19 times in RAW264.7 cells, and 4.12 and 4.31 times in CD11b^+^ bone marrow cells more than control individually by the qPCR analysis (P < 0.01). Neurokinin B (NKB) is a neuropeptide in the tachykinin family that acts as a neurotransmitter and neuromodulator in the sensory nerve system and has a high affinity for tachykinin receptor 3 [34]. Previous study indicated that NKB was expressed by the peripheral bone tissue and sensory nerve in bone [32]. In oral squamous cell carcinoma invaded bone destructed site, SHH from tumor cells might upregulate the tachykinin receptor 3 expression in the preosteoclasts and consequently stimulate osteoclasts differentiation indirect manner.

In conclusion, our results showed that SHH and its signaling molecules were frequently expressed in the tumor bone microenvironment in mandible resected specimens of oral squamous cell carcinoma, and SHH stimulated the differentiation and activation of osteoclasts directly. These findings strongly suggest that the SHH pathway plays an important role in the bone destruction in oral squamous cell carcinoma and should be considered a potential therapeutic target in the future.

## Supporting Information

S1 Fig Key pathways predicted by Single Experiment Analysis from the gene list including factors that were increased or reduced more than two-fold following treatment with SAG in RAW264.7 cells. (TIF)

S2 Fig List of selected genes that belong to the top 3 pathways indicated in S1 Fig and were changed more than three times. The color range corresponds to the value of fold change.
(TIF)

S3 Fig CD11b^+^ bone marrow cells were isolated from floxed Smoothened mice, infected with Cre recombinase encoding adenovirus (Ad-Cre) or control GFP adenovirus (Ad-GFP), maintained in differentiation medium for three days, and then treated with SHH. TRAP-positive multinucleated cells (nuclear number > 3) were counted as osteoclasts. The data from a typical experiment are presented. Data are shown as the mean ± SD. Statistically significant differences (**P < 0.01) between the indicated groups are marked by asterisks.
(TIF)

S4 Fig (**A**) Detection of p-ERK, ERK, p-p38, and p38 by immunoblot analysis after RANKL (50 ng/ml) in murine macrophage CD11b^+^ cells with or without SHH (500 ng/ml). (**B**) qPCR analysis of NFATc1 in CD11b^+^ cells 24 h after RANKL (30 ng/μl) with or without SHH (500 ng/ml) treatment.
(TIF)

S5 Fig Microarray information. (XLS)

## Abbreviations

TRAP: tartrate-resistant acid phosphatase
RANKL: receptor activator for nuclear factor κ B Ligand
