# Supplementary figures and images for: The Role of Sonic Hedgehog Signaling in Osteoclastogenesis and Jaw Bone Destruction
Source: PLoS One. 2016 Mar 23;11(3):e0151731. doi: 10.1371/journal.pone.0151731 (PMC4805186; doi:10.1371/journal.pone.0151731)

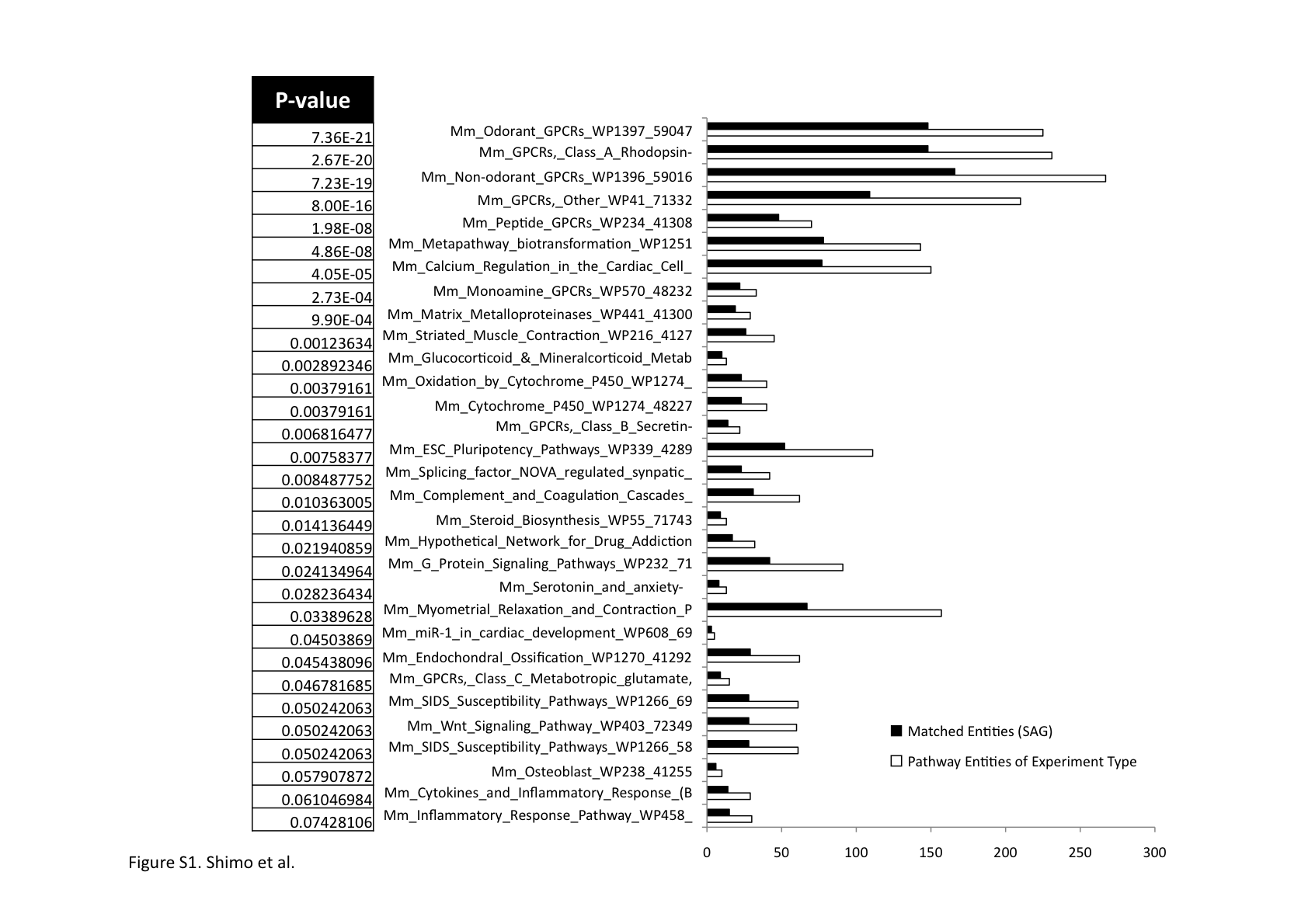

Supplement: S1 Fig — (TIF) [file pone.0151731.s001.tif]

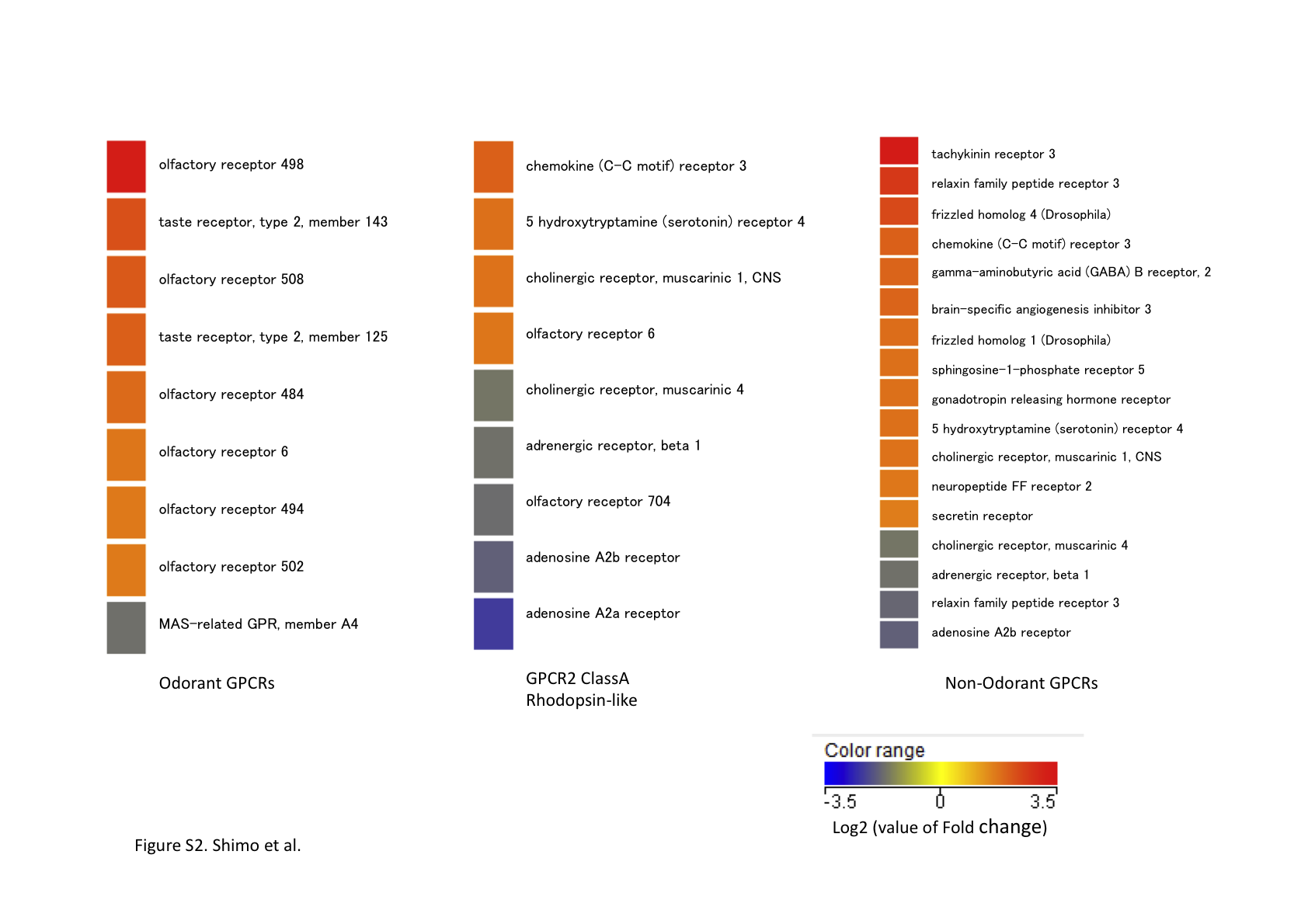

Supplement: S2 Fig — The color range corresponds to the value of fold change. (TIF) [file pone.0151731.s002.tif]

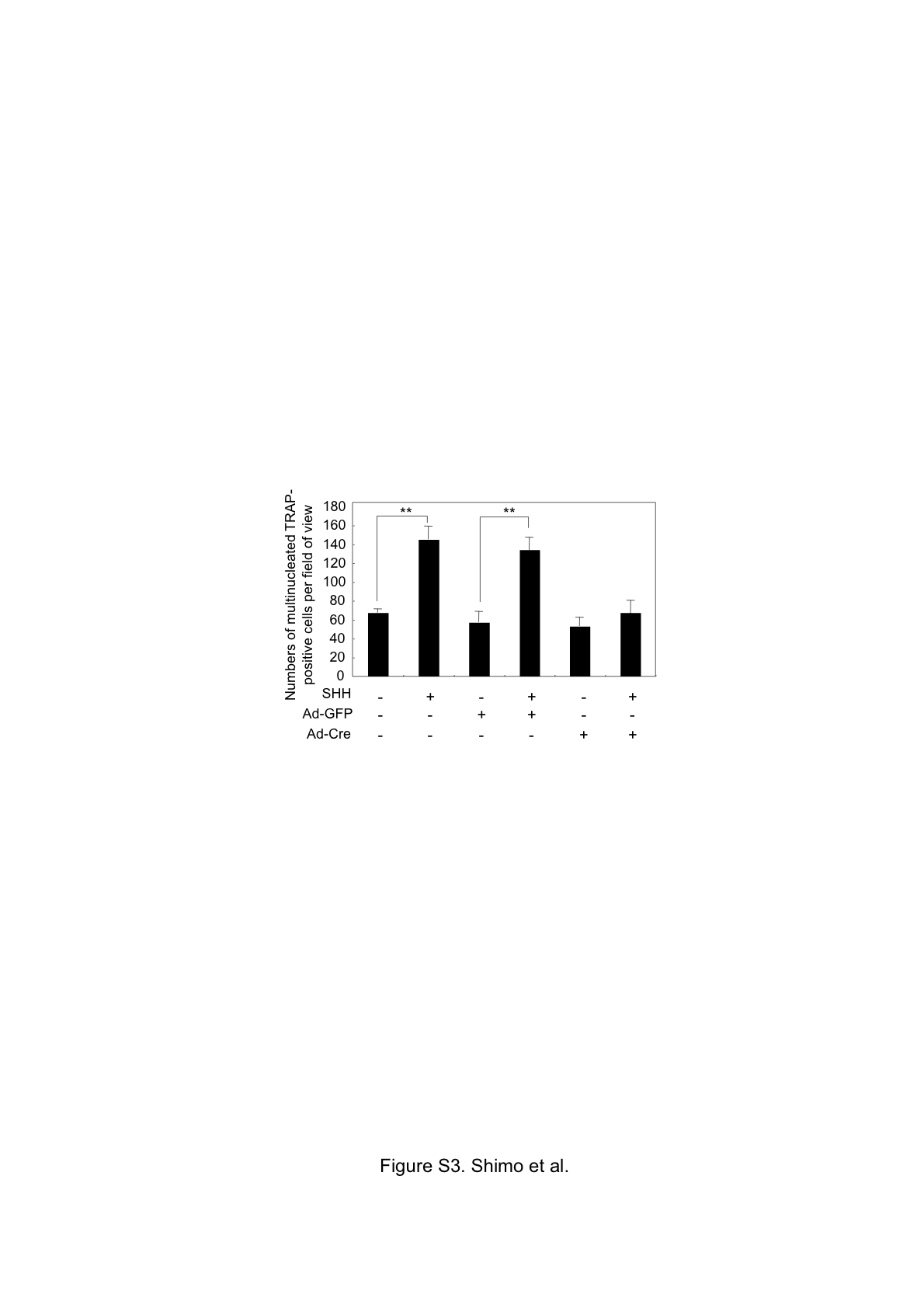

Supplement: S3 Fig — TRAP-positive multinucleated cells (nuclear number > 3) were counted as osteoclasts. The data from a typical experiment are presented. Data are shown as the mean ± SD. Statistically significant differences (**P < 0.01) between the indicated groups are marked by asterisks. (TIF) [file pone.0151731.s003.tif]

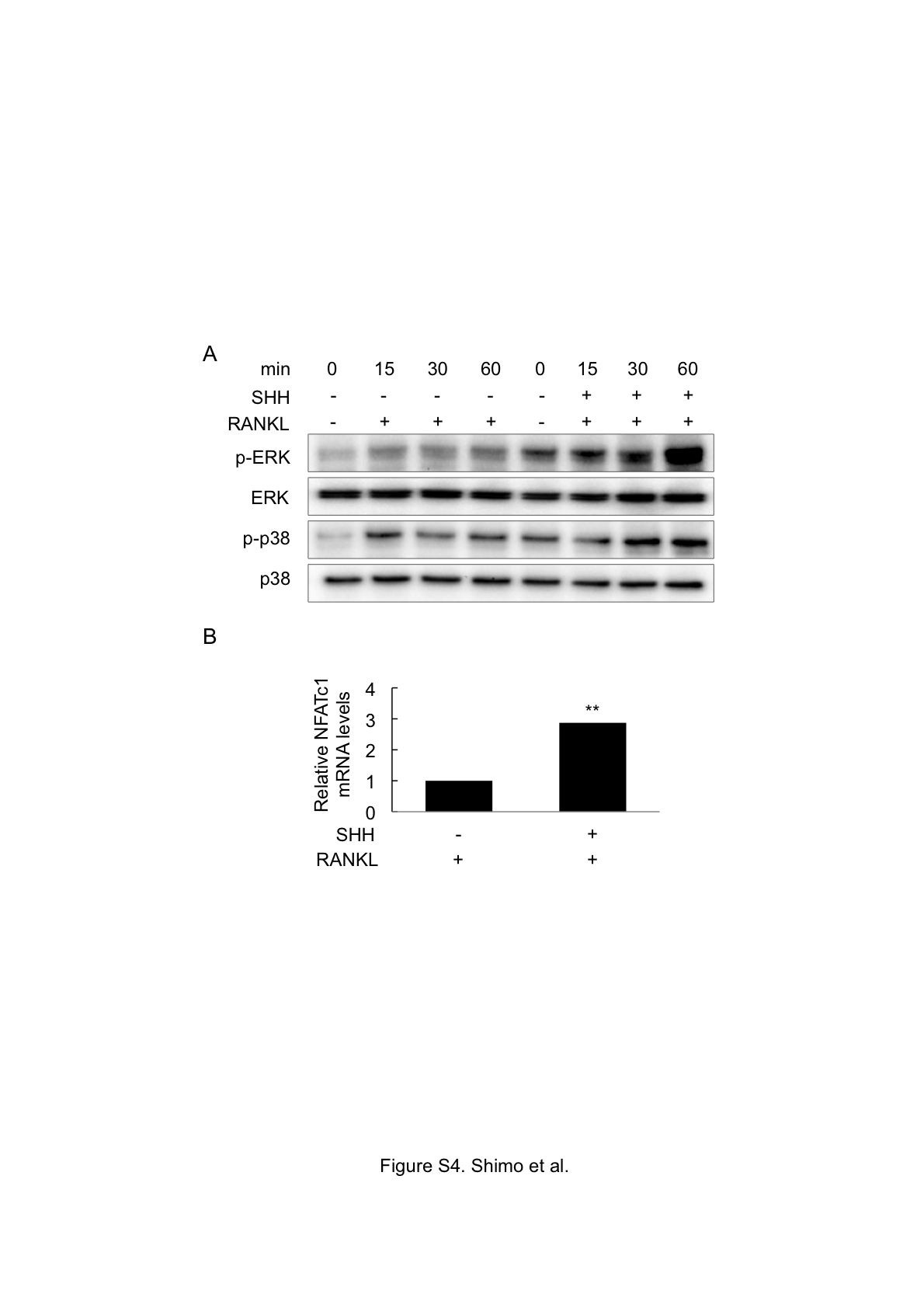

Supplement: S4 Fig — (A) Detection of p-ERK, ERK, p-p38, and p38 by immunoblot analysis after RANKL (50 ng/ml) in murine macrophage CD11b+ cells with or without SHH (500 ng/ml). (B) qPCR analysis of NFATc1 in CD11b+ cells 24 h after RANKL (30 ng/μl) with or without SHH (500 ng/ml) treatment. (TIF) [file pone.0151731.s004.tif]
